# Unveiling the Composition of La Pajarita PVAc-Based Paints in Joan Miró’s Studio and in Three Artworks from the 1970s

**DOI:** 10.3390/polym16223146

**Published:** 2024-11-12

**Authors:** Mar Gomez Lobon, Enric Juncosa Darder, Carlos Palomino Cabello, Marta Bauza, Francesca Caterina Izzo

**Affiliations:** 1 Independent Researcher, Artco Services, 07110 Mallorca, Spain; 2 Conservation Department, Fundació Pilar i Joan Miró a Mallorca, 07015 Palma de Mallorca, Spain; enric.juncosa@miromallorca.com; 3 Chemistry Department, Universitat de les Illes Balears, 07122 Palma de Mallorca, Spain; carlos.palomino@uib.es (C.P.C.); marta.bauza@uib.cat (M.B.); 4 Heritage and Conservation Science, Department of Environmental Sciences, Informatics and Statistics, Ca’ Foscari University of Venice, 30123 Venezia, Italy

**Keywords:** Joan Miró, La Pajarita, Fundació Miró Mallorca, artist studio, PVAc, vinyl paints, artist materials, 20th century artworks, latex paints, Py–GC–MS

## Abstract

In this study, we present the first characterisation of the polyvinyl acetate (PVAc) paints of commercial Spanish brand La Pajarita preserved in the studios of Joan Miró (1893–1983) in Mallorca, Spain. Investigation of several black and white paint samples using complementary analytical techniques (XRD, SEM–EDX, FTIR, and Py–GC–MS) allowed for the identification of pigments and binding media in studio materials, as well as in three artworks dating from the 1970s. Through comparative analysis, it was possible to find significant similarities between the composition of La Pajarita paints conserved in cans in the artist’s studio with black and white painted layers from three artworks by Miró. Miró’s use of La Pajarita paints is extensively documented in studio notes, photographs, and videos, and these paints are known to have been used by other significant Spanish artists. However, their composition has remained largely undiscovered until now. This research contributes to the knowledge of PVAc paints, providing evidence of their use by Joan Miró. The analytical results serve as a valuable reference for comparing and identifying these synthetic paints in other artworks, as well as aiding in attribution or dating studies. Furthermore, the study demonstrates the significance of materials found in artists’ studios as a fundamental resource for identifying the materials present in artworks.

## 1. Introduction

Joan Miró i Ferrà was born in 1893 in Barcelona and died in Mallorca in 1983. Miró was one of the most ground-breaking and influential artists of the 20th century. Working and experimenting in every known medium, he left an enormous legacy of work, which includes his studios in Mallorca, where he lived and worked from 1956 until his death.

Miró’s studios in Mallorca, Taller Sert, built in 1956 [1], and Son Boter, an adjacent property he purchased in 1959 [2], represented for Miró the realisation of his dream to have a large studio, and from this time he started working on large formats and achieved a period of maximum freedom of expression. Miró’s studios in Mallorca have been preserved as the artist left them at the time of his death and are now part of the Fundació Miró Mallorca, presenting an invaluable resource for the study of the artist’s materials and techniques.

The documentation of materials in artists’ studios has been the subject of various recent studies [3,4,5,6,7,8,9,10], which contribute to building a collective archive of historical painting materials. This archive can serve as a valuable reference for identifying the composition of the paints used by artists in their works, helping to better understand their behaviour over time (drying, ageing, and degradation). The materials preserved at artists’ ateliers can also help with the study of conservation issues observed in their artworks, and previous scientific analysis of paints preserved in Miró’s studios have helped in understanding the deterioration observed in his paintings today [11].

Miró’s artistic stage in Mallorca coincided with the introduction of acrylic paints for artists’ use, and he was possibly one of the first artists to introduce water-based emulsion paints in the 1960s, a new medium that offered a range of new possibilities for artists [12].

Water-based emulsion paints were developed in the mid-1950s in the United States, and by the mid-1960s, many product lines were being offered by artist paint companies in Europe and worldwide [12,13,14], thus marking a significant transformation in the art world. Acrylic emulsion paints, often referred to as ‘acrylics’, were marketed to contemporary artists, with Andy Warhol and Helen Frankenthaler amongst the first to use them [13].

Miró was attracted to this new water-based medium, since it offered new possibilities, such as quicker drying and different viscosity and working properties. In an interview in 1974, he mentioned that he sometimes used acrylics because of their rapid drying properties, but he also appreciated the richness that could be obtained with the ‘slow-drying’ oil colours [15].

What are commonly referred to as ‘acrylic paints’ include paints that have acrylic resins as paint binders, but also polyvinyl acetate or PVAc-based paints. PVA, synthesised by Fritz Klatte in 1912 for the first time, results from the free-radical polymerization of vinyl acetate monomer units. Based on temperature reactions and the different typology of initiators, catalysts, and solvents used, the properties of the final PVA polymers may differ [13]. Initially, the principal application of PVA was as an adhesive, but from the 1930s, PVA-based waterborne resins were introduced to the market as house paint [13,16]. While acrylic emulsion paints became the most popular formulation in America, PVAc made an impact in the artistic field in the late 1940s, and it has remained the main type of binder for house paint in many European countries, commonly referred to as ‘vinyl paints’ [13]. Although PVAc paints were never as popular as acrylic emulsion paints (since they were considered to be of lower quality), they were still used by many notable artists, including both household and artist-quality vinyl emulsion paints [16].

It is possible that Miró had seen the use of these new water-based emulsion paints by American abstract expressionists such as Mark Rothko in his trip to United States in 1959, which inspired him to try them. In any case, Miró’s *Catalogue Raisonné* [17] lists a series of four paintings from 1960 as acrylic and pastel crayon on canvas, and another series of five paintings, also from 1960, as oil and acrylic on canvas (Cat. No. 1128-1132 [17]). His first mention of these new water-based paints of the brand La Pajarita is also from 1960 [18].

La Pajarita, founded in Valencia, Spain, claims to have developed the first plastic paint in Europe in 1954 and continues its production of vinyl paints for artists today (https://www.lapajarita.es/wp-content/plugins/gtranslate/url_addon/gtranslate.php?glang=en&gurl=About-us/ (accessed on 28 October 2024)). Miró continued to mention this brand through to the 1970s in many of his notes preserved at the Fundació Miró in Barcelona [18] and at the Fundació Miró Mallorca, and three cans of La Pajarita black paint are preserved in Taller Sert.

Although the first commercial polyvinyl acetate paints, including artist-grade lines, were introduced in the 1950s, knowledge of their properties and degradation processes remains relatively limited compared to acrylic emulsion paints [16]. In fact, while for different types of acrylic polymers, the ageing pathways are numerous and generally well known, for PVAc homopolymers, the main degradation pathway is scission, which can have a direct impact on the mechanical behaviour of PVAc-painted surfaces [19,20,21,22,23].

While studies into the composition and behaviour of other contemporary brands of vinyl paints available in neighbouring countries, such as the vinyl paints produced in Portugal by Favrel [23,24] or the Flashe vinyl paints produced by Lefranc Bourgeois [25], have been carried out, the Spanish La Pajarita vinyl paints and their use by 20th-century artists remains largely undiscovered until now. In fact, it should be noted that in a recent review of the history and formulation of polyvinyl acetate paints [16], the brand La Pajarita is not mentioned.

Hence, these PVAc paints, which according to the manufacturers were used not only by Miró but also by other significant artists such as Salvador Dalí and Antoni Tàpies, remain largely unexplored, as is the development of modern paints in Spain and their adoption by artists during the 60s and 70s, a time of limited exchange with other European countries (Spain was under the Francoist dictatorship from 1936 until 1975. During these years imported materials would have been limited and expensive, and hence artists would have relied mainly on available local materials and brands). Research into their composition would be of interest to understand the behaviour and ageing of artworks created with these paints. Knowledge of the materials constituting paintings is also crucial in order to design appropriate conservation treatments.

In this study, which is part of a wider project to investigate Joan Miró’s pigments during his stage in Mallorca [26], we present the results of the first characterization of La Pajarita paints preserved in the studio of Joan Miró. The aims of the study were to unveil the composition of these commercial paints, both in terms of organic and inorganic compounds, pigments, additives, etc., and to determine their presence as artistic materials in real artworks, using the studio materials as a reference.

## 2. Materials and Methods

### 2.1. Samples from Studio Materials and Artworks

**Figure 1:**
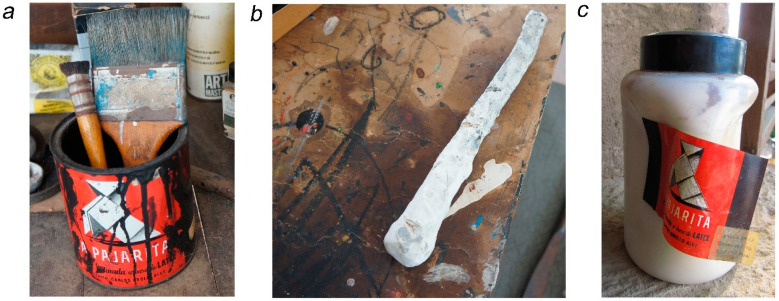
Studio materials sampled. (**a**) Can of La Pajarita black paint (sample N1b). (**b**) Mixing stick covered with white paint (sample B3). (**c**) Bottle of La Pajarita white paste (sample B6).

**Figure 2:**
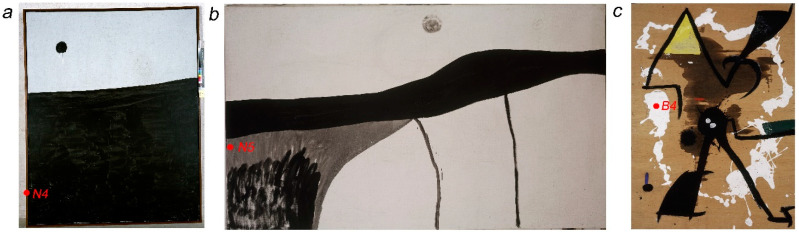
Artworks by Joan Miró sampled, with location of samples. (**a**) Untitled (FPJM-00106), 1973, acrylic and charcoal on canvas, 216 × 173.8 cm (sample N4). (**b**) Untitled (FPJM-00108), 1973, acrylic and charcoal on canvas, 174 × 293 cm (sample N5). (**c**) Untitled (FPJM-00135), 1977, oil and acrylic on plywood, 122 × 89 cm (sample B4).

Six representative samples of black and white paints were taken from Miró’s studio materials (Figure 1) and three artworks (Figure 2) to investigate their composition. The details of the samples are also included in Table 1.

#### 2.1.1. Black Paint Samples

A sample of black paint was taken from one of the cans of La Pajarita conserved in Taller Sert (Figure 1a). The can is labelled ‘Latex-based satin paint’ and it is open, with remnants of black paint on the inside. The sample was taken from one of the paint drips on the sides (sample N1b, see Table 1).

Other 2 microsamples of black paint were taken from the large-format paintings Untitled (FPJM-00106) (Figure 2a) and Untitled (FPJM-00108) (Figure 2b) in the collection of the Fundació Miró Mallorca, labelled N4 and N5, respectively, in this study.

These artworks were both painted by Miró in 1973 and are catalogued as ‘acrylic and charcoal on canvas’. The flat and fluid appearance of the black paint in both paintings, with dripping paint observed in some areas, is consistent with that of water-based emulsion paint, and very similar in colour and appearance to the remains of black paint in the cans of La Pajarita preserved in Taller Sert.

#### 2.1.2. White Paint Samples

In the painting Untitled (FPJM-00135), 1977, also belonging to the collection of the Fundació Miró Mallorca, Miró used white paint with a very plastic-like appearance, thickly applied directly over the plywood support (Figure 2c). The work was formerly framed with the glass in contact with the paint, which caused some of the paint to adhere to the glass. When the work was reframed, the remains of paint adhered to the glass were preserved, and these have been used for analysis (sample B4—see Table 1). This paint resembles the white paint that covers a wooden stick used by Miró to stir paint kept in Taller Sert (Figure 1b), which was also sampled (sample B3) to find out if this paint was the same as that in the artwork and if it belonged to the brand La Pajarita.

Finally, a sample of the liquid white paste inside a plastic bottle of La Pajarita in Son Boter, also labelled ‘Latex-based satin paint’, was also taken (sample B6). The liquid exhibited a consistency more similar to an adhesive than a paint and presented a phase separation (with a white phase and a transparent one).

### 2.2. Analytical Techniques

Both the inorganic and organic fraction of the selected samples were thoroughly investigated by means of a tried-and-tested multi-analytical approach able to unveil the composition of pigments, binders, fillers, additives, etc. in commercial paints.

#### 2.2.1. Scanning Electron Microscopy with Energy Dispersive X-Ray Analysis (SEM–EDX)

Elemental analyses were performed on all samples with a scanning electron microscope (SEM, Hitachi S-3400N, used at 15 kV), equipped with a Bruker AXS XFlash 4010 EDS system.

#### 2.2.2. X-Ray Diffraction (XRD)

Identification of crystalline phases was performed through X-ray diffraction analysis on all samples using a Bruker D8 Advance diffractometer with monochromatized CuK_α_ radiation (1.54 Å) and using a one-dimensional LynxEye detector. Powder diffraction continuous data were collected at 40 kV between 10° and 50° with a step size of 0.02° and a time step of 2 s.

#### 2.2.3. Fourier-Transform Infrared Spectroscopy (FTIR)

FTIR analyses were performed on raw samples in ATR mode with a Bruker Tensor 27 system in the spectral range 4000 cm^−1^ to 750 cm^−1^, with 3 cm^−1^ of resolution and accumulating 32 scans.

#### 2.2.4. Pyrolysis Gas Chromatography–Mass Spectrometry (Py/GC–MS)

The composition of the organic fraction of samples was analysed using thermally assisted hydrolysis and methylation and pyrolysis gas chromatography–mass spectrometry (THM–Py–GC/MS).

According to previous studies on synthetic binding media in 20th-century artworks [27,28,29,30,31,32,33,34], different methodologies were tested: flash pyrolysis or double-shot pyrolysis, with or without methylation pretreatment with tetramethylammonium hydroxide (TMAH), 25%, in methanol. The experimental tests, performed on 30 μg of each sample placed in eco-cup pyrolysis crucibles, highlighted that the best conditions for the selected paint samples were to analyse them without pretreatment and opt for a pyrolysis oven (high speed) temperature range starting at 360 °C and raised to 700 °C at a rate of 500 °C/min for a total time of 1 min.

The pyrolysis unit used was a Frontier Lab 3030D, micro furnace pyrolyser mounted on a Thermo Scientific Focus GC/ISQ mass spectrometer combination. Separation took place on a SLB5 ms (Supelco) column with a length of 20 m, an internal diameter of 0.18 mm and a film thickness of 0.18 μm. Helium was used as the carrier, with a constant flow of 0.9 mL/min. The split ratio was 1:30. The temperature program used was 35 °C, stable for 1 min, subsequently raised by 60 °C per minute until 110 °C, raised by 14 °C per minute until 240 °C, and by 5 °C per minute until 315 °C, then held stable for 2 min. The temperature of the interface was set at 250 °C, the temperature of the ion source at 220 °C. Mass spectra were recorded from 29 until 600 amu with a speed of 5 scans per second. Xcalibur 2.1 software was used to collect and process the mass spectral data.

The interpretation of the results was conducted using the ESCAPE system, an expert system for characterization of (THM–)Py–GC/MS data using AMDIS 2 and Excel [35]. The ESCAPE AMDIS library is based on spectra from the NIST library and data shared by experts in the conservation science field.

## 3. Results and Discussion

The results of the characterisation of the samples are summarised in Table 1. Data are reported for each analytical technique used, in order to highlight the major results obtained.

In the following paragraphs, the discussion of the data considers separately the identification of the synthetic binder and various organic additives and the detection of black and white pigments and different inorganic compounds in the paint samples, thus providing a concise and precise description of the experimental results, their interpretation, and the conclusions that can be drawn.

### 3.1. Identification of the Synthetic Binding Media

Considering that an important focus of this study is to provide for the first time information about the composition of La Pajarita’s synthetic paints, samples from the studio materials and Miró’s paintings were all subjected to Py–GCMS analysis, providing an answer to this important piece of knowledge.

**Figure 3:**
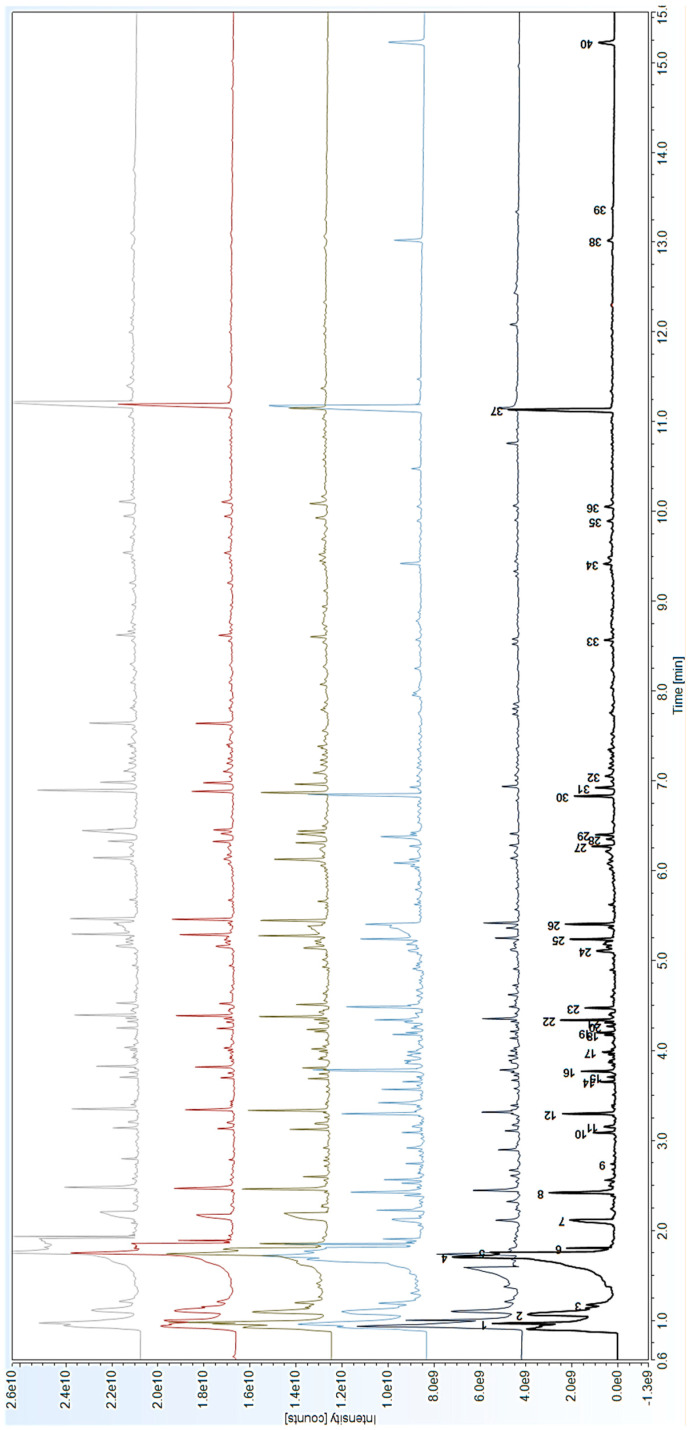
Pyrograms of samples N1b (black), B4 (dark grey), N4 (light blue), B6 (light green), B3 (red) and N5 (grey) by means of Py–GC–MS analysis. For peak identification, refer to peak numbers of Table 2.

Figure 3 depicts the pyrograms of the N1b, N4, N5, B3, and B4 samples. The presence of a PVAc-based paint was verified in all the analysed samples. The typical pyrolysates of PVAc, such as acetic acid, acetone, 1,3-cyclopentadiene, benzene (and benzene derivatives), toluene, indane, indene, methylindenes, and naphthalene (and its derivatives), which have been reported in the literature [32,33], were detected.

Acetic acid presented the most intense peak, as expected from the C-O bond’s pyrolytic decomposition from the side chain and the main backbone of PVAc. In addition to acetic acid, pyrolytic decomposition can yield aromatic hydrocarbons through decomposing the polymer backbone and cyclizing olefin intermediates [36].

All the identified compounds are reported in Table 2.

The PY–GC–MS analysis detected the presence of different additives in the paints, specifically:

− Phthalate-based compounds, such as dibutyl phthalate, butyl 2-ethylhexyl phthalate, bis(2-ethylhexyl) phthalate, and butyl dodecyl phthalate, added in the past as external plasticisers;
− Benzoate esters used as coalescing agents, such as butyl benzoate and 2-ethyl-hexyl benzoate;
− Antifoam agents, polydimethylsiloxane and silicon oils in particular.

The significant presence of external plasticisers is associated with the physical plasticising method (i.e., external plasticising) that is actually easier to produce and use, but on the other hand leads to more enhanced migration and volatility of plasticisers.

The issue of their migration is as important as ever, since the loss of plasticisers can significantly alter the mechanical properties of pictorial surfaces.

**Figure 4:**
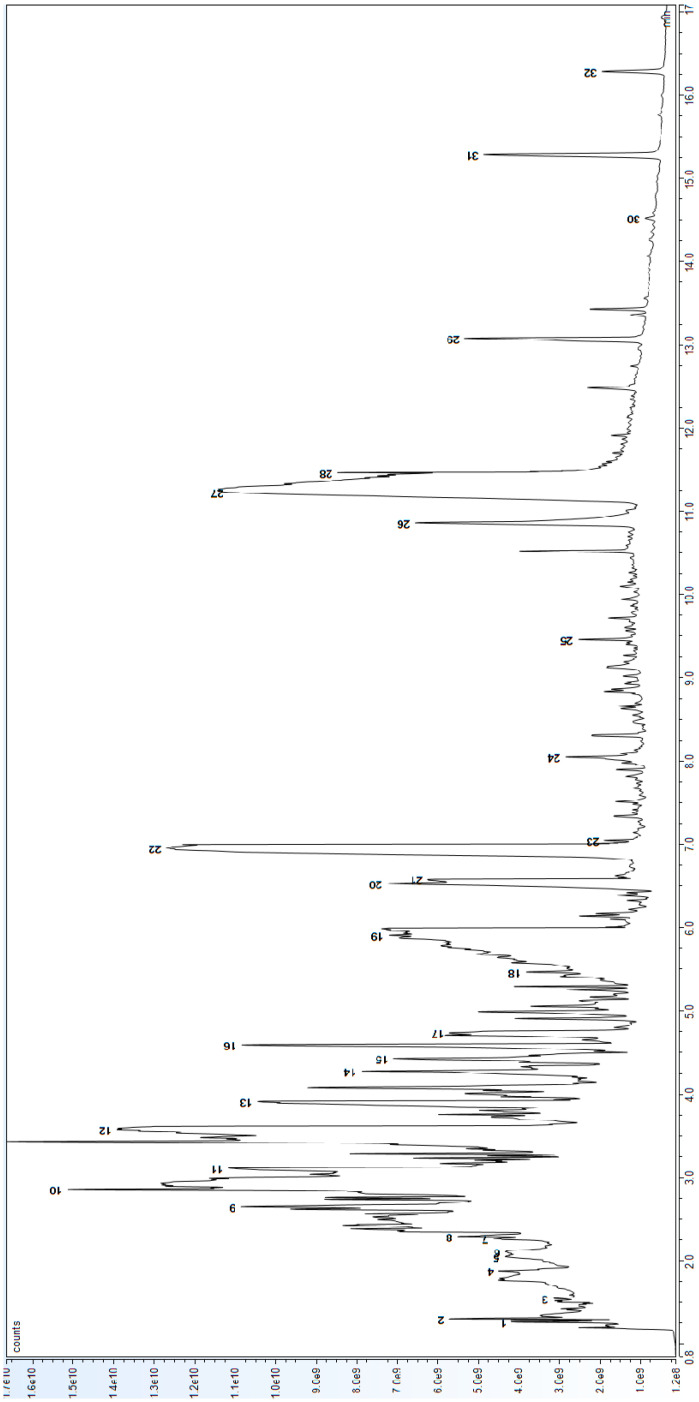
Pyrograms of sample B6 (liquid paint) by means of Py–GC–MS analysis. For peak identification, refer to peak numbers of Table 3.

The Py–GC–MS analysis of the liquid B6 sample taken from a jar of La Pajarita paint deserves a separate discussion. Figure 4 shows the pyrogram obtained, which shows, in addition to the many compounds already highlighted in Figure 3 and Table 2, some important differences. Table 3 shows the list of pyrolysates detected within the liquid phase of the commercial paint.

Clearly, all the components linked to the solvent fraction are identified (such as propene, butene, benzene, etc.), but it is very interesting to note the wealth of additives, including:

− External plasticisers (and one notices other types of phthalates from those highlighted earlier);
− Antifoam agents (such as siloxanes);
− Freeze–thaw agents (such as ethylene glycol);
− pH buffers (such as ammonia acetate).

The identification of phthalic acid in the liquid paint could be related to the degradation of phthalates due to hydrolysis, photodegradation, and/or biodegradation [36]. These pathways are generally more pronounced in the atmosphere, but considering the state of preservation of the bottle, it is most likely that the material inside has reacted with oxygen, light, and temperature, thus leading to the degradation of the original material.

Finally, the composition of La Pajarita paints was also corroborated with the company’s technical department. According to the information provided, a vinyl binder has always been used for artist paints, and it is still used today, but many of the additives used in the 70s, such as phthalates, have been modified or eliminated over the years due to safety regulations, since they are now considered harmful to health and the environment. According to the company’s technical department, the vinylic dispersions used have evolved to adapt to safety and environmental regulations. For example, the dispersions Mowilith D025 and Mowilith DVB and DVB017 used in the 1980–1990s contained diisobutyl phthalate (DIBP), which is nowadays banned (correspondence with technical department, 18 November 2021).

### 3.2. Pigments and Additives in Black Paint Samples

The pigment analysed in La Pajarita paint can (sample N1b) and in the samples of black paint from the two artworks from 1973 (samples N4 and N5) was identified as carbon black, considering the black colour, the absence of characteristic elements detected on SEM–EDX (apart from C and O), and on XRD there being no trace of graphite or other black pigment.

**Figure 5:**
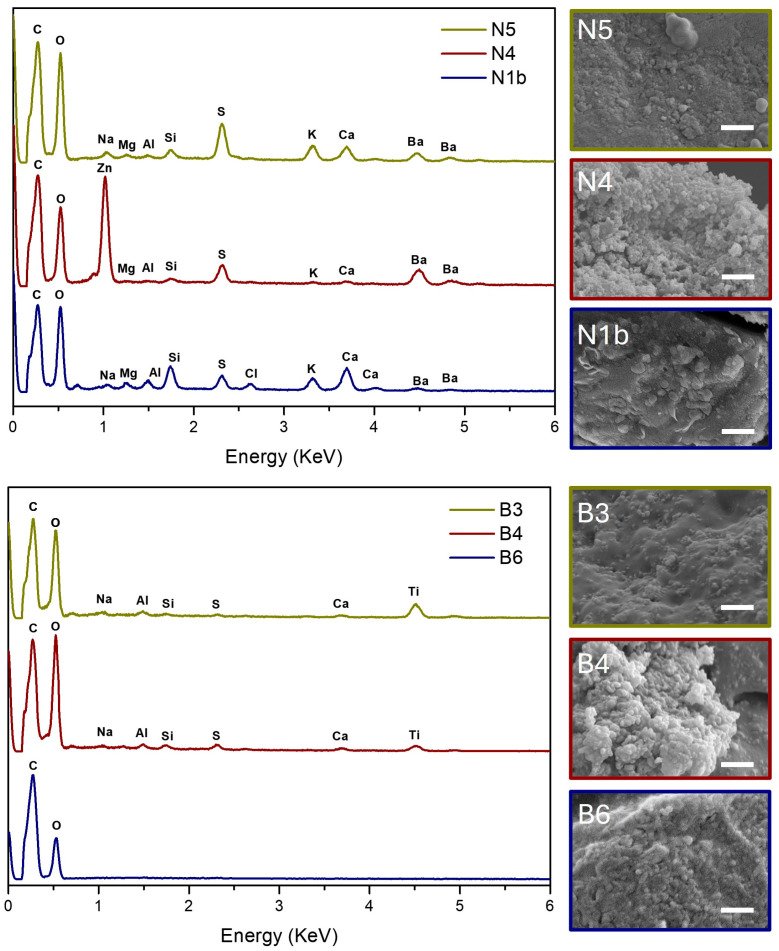
EDX and SEM images of (**up**) black samples and (**below**) white samples. Scale bar 2 μm.

Carbon black is a pigment composed of pure carbon, and is obtained by collecting the soot produced by burning oils and fats [37]. According to information provided by La Pajarita, the pigment used for this line of black paints has always been carbon black (correspondence with technical department, 18 November 2021). The elemental analysis (Figure 5 (up)) was very similar in all samples, coinciding with, apart from carbon, the elements identified in smaller quantities (Mg, Si, S, K, Al, Ba, and Ca). These elements are associated with the presence of inorganic additives, fillers, and extenders.

**Figure 6:**
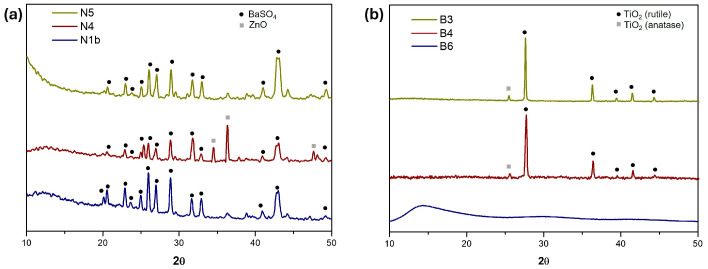
XRD patterns of (**a**) black samples and (**b**) white samples.

Barium sulphate (barite), a common filler in industrial paints, was also identified in the three black samples with XRD analyses, while zinc white (ZnO) was detected in the work Untitled FPJM-00106 (sample N4), as shown in Figure 6a; however, this pigment may be due to a contamination of the preparation layer.

**Figure 7:**
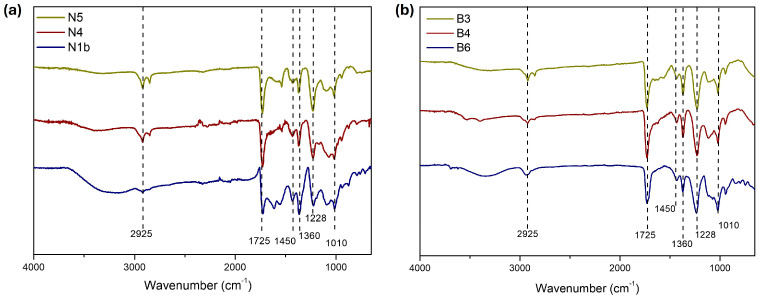
FTIR of (**a**) black samples and (**b**) white samples.

The FTIR spectra of the black samples (Figure 7a and Table 1) showed similar bands at about 2925, 1725, 1450, 1360, 1228, and 1010 cm^−1^, which are attributed to -CH, C=O, -CH_2_, -CH_3_, and -C-O vibrations, corresponding to the binder present in the samples. These main bands correspond with the spectral markers 2933 (w), 1728-30 (vs), 1371 (m), 1434, 1371 (m), 1224 (vs), and 1019 (s) reported for PVAc in the literature [38,39]. FTIR spectra of black samples also showed some of the bands reported for barium sulphate at 635 (w) and 600 (s), and dolomite (magnesium and calcium carbonate) at 1432 (m), 875 (w), and 793 (w) [40].

Given the similarity in the results of the analysis of all the components of the paint, including the plasticisers and other additives, we can conclude that Miró used black paint of the brand La Pajarita (from the can kept in the studio or another similar one) in the works Untitled (FPJM-00106) and Untitled (FPJM-00108). Since the artwork Untitled (FPJM-00106) is a part of a tryptic (with the artworks Untitled FPJM-00109 and Untitled (FPJM-00110 [41]), it is very likely that Miró also used this paint for the other two works, although this could be confirmed only by additional analysis.

### 3.3. Pigments and Additives in White Paint Samples

Titanium dioxide in the rutile structure was identified with XRD (Figure 6b) in the artwork Untitled (FPJM-00135) (sample B4) and in the paint-mixing stick (sample B3), confirming that the pigment in both samples was titanium white. In the case of the liquid sample from La Pajarita jar (sample B6), no crystalline compounds were detected (Figure 6b), as expected. SEM–EDX analysis (Figure 5 (below)) of samples B3 and B4 confirmed the presence of Ti and O, with additional detection of minor elements Na, Al, Si, S, and C in both samples, indicating their similarity. In contrast, sample B6 showed detection only of C and O, considering the main organic fraction in it.

The FTIR spectra of the three analysed white samples (Figure 7b and Table 1) show practically the same bands for the PVAc binder as those in the black paint samples. The spectra of samples B3 and B4 have the additional band of 1116(s), which can also be assigned to the binder [38], as well as the broad absorption for titanium white pigment between 432–800 cm^−1^.

Given the similarity of the analysis results, it can be concluded that the white paint on the stirring stick in Taller Sert (sample B3) is the same as the white paint used by Miró in the artwork Untitled (FPJM-00135) (sample B4). The composition of the binder also coincides with that identified in La Pajarita black paint (sample N1b). According to the information provided by the brand, the pigment used in the 1970s was titanium dioxide produced by the sulphate process with a composition similar to pigment Tioxide TR92 (correspondence with technical department, 18 November 2021), so although no cans of white paint were available to sample, it is highly likely that the white paint in this artwork is also from La Pajarita brand.

Titanium dioxide rutile and carbon black have been proved to stabilise this type of polymer [36], which could explain the good condition of the artworks examined.

In sample B6 from the liquid inside plastic bottle, no pigment was detected, hence the material appears to contain only the binder identified with Py–GCMS (see Figure 4 and Table 3). Thanks to the code on the label (‘article 27’), this product was identified in an old catalogue as an adhesive paste, intended to be mixed with pigments or paint to obtain reliefs. Therefore, it can be deduced that the binder used in La Pajarita paints is the same as that of other products such as this adhesive paste.

## 4. Conclusions and Further Work

This study presents the first characterisation of La Pajarita PVAc paints, a brand of vinyl paints used by many important Spanish artists during the 1960s and 1970s, and still used by artists today. The analysis identified the binder and additives present in the paints sampled, as well as the pigments and extenders. The study has also shed some light on the complexity of PVAc paints and the phthalate-based compounds and coalescing and antifoam agents added to these paints in the 1970s.

The composition of La Pajarita’s binder, a polyvinyl acetate (PVAc) homopolymer, differs from other well-known brands of vinyl paints such as Flashe that are based on emulsions of polyvinyl acetate/vinyl versatate (PVA/VeoVA) [25], which, together with ethylene vinyl acetate copolymer (p(E-co-VAc)), are the three more common types of VAc-based polymers [34].

Through comparative analysis, it has been possible to confirm that Miró used this brand of paint in the black paint in the artworks Untitled (FPJM-00106) and Untitled (FPJM-00108), painted in 1973, based on similarities in pigment composition and proportions of vinyl resin, phthalates, and other organic components. The composition of the binder was also used to identify this brand of paint in the white paint in the artwork Untitled (FPJM-00135), even though it was a different colour.

Miró’s use of La Pajarita paints is widely documented in his studio notes, as well as in photographs and videos. Through this study, the use of this brand of paint in his paintings has been confirmed by providing evidence through scientific analysis.

The results of the analyses and the characterisation of the binder are of great value, since they can be used as a reference for the identification of this brand of PVAc paint in other artworks by Miró, as well as in works by other 20th-century artists, helping with dating and attribution studies, as well as with the conservation of artworks.

The authors expect to carry out further investigation into the properties and composition of La Pajarita paints through the analysis of colour charts and other cans of paint that have been found in Miró’s studio since the completion of the project.

## Figures and Tables

**Table 1:** Summary of main results obtained from characterisation of samples.

	No.	Image	Object	SEM–EDX	XRD	FTIR (ATR)	PY–GC–MS (Main Compounds Detected)	Interpretation
**BLACK PAINT SAMPLES**	N1b	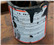	Can of black paint, La Pajarita, Taller Sert	C, O, S, Ba (Na, Mg, Si, K, Al, Ca, Cl)	BaSO_4_	PVAc BaSO_4_ CaMg(CO_3_)_2_	acetic acid, acetone, 1,3-cyclopentadiene, benzene (and benzene derivatives), toluene, indane, indene, methylindenes, naphthalene; dibutyl phthalate, butyl 2-ethylhexyl phthalate, bis(2-ethylhexyl) phthalate and butyl dodecyl phthalate; butyl benzoate and 2-ethyl-hexyl benzoate	Pigment: carbon black Fillers/extenders: barium sulphate, dolomite Binder: polyvinyl acetate-based paint with external plasticisers (dibutyl phthalate, butyl 2-ethylhexyl phthalate, bis(2-ethylhexyl) phthalate and butyl dodecyl phthalate), coalescing agents (butyl benzoate and 2-ethyl-hexyl benzoate) and other additives
	N4	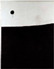	Untitled (FPJM-00106)1973 Catalogued as acrylic and charcoal on canvas 216 × 173.8 cm	C, O, S, Ba, Zn (Mg, Si, K, Al, Ca)	BaSO_4_ ZnO	PVAc BaSO_4_ CaMg(CO_3_)_2_	acetic acid, acetone, 1,3-cyclopentadiene, benzene (and benzene derivatives), toluene, indane, indene, methylindenes, naphthalene; dibutyl phthalate, butyl 2-ethylhexyl phthalate, bis(2-ethylhexyl) phthalate and butyl dodecyl phthalate; butyl benzoate and 2-ethyl-hexyl benzoate	Pigment: carbon black, zinc oxide possibly corresponds to priming layer Fillers/extenders: barium sulphate, dolomite Binder: polyvinyl acetate-based paint with external plasticisers (dibutyl phthalate, butyl 2-ethylhexyl phthalate, bis(2-ethylhexyl) phthalate and butyl dodecyl phthalate), coalescing agents (butyl benzoate and 2-ethyl-hexyl benzoate) and other additives
	N5	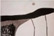	Untitled (FPJM-00108) 1973 Catalogued as acrylic and charcoal on canvas 174 × 293 cm	C, O, S, Ba (Na, Mg, Si, K, Al, Ca)	BaSO_4_	PVAc BaSO_4_ CaMg(CO_3_)_2_	acetic acid, acetone, 1,3-cyclopentadiene, benzene (and benzene derivatives), toluene, indane, indene, methylindenes, naphthalene; dibutyl phthalate, butyl 2-ethylhexyl phthalate, bis(2-ethylhexyl) phthalate and butyl dodecyl phthalate; butyl benzoate and 2-ethyl-hexyl benzoate	Pigment: carbon black Extenders: barium sulphate, dolomite Binder: polyvinyl acetate-based paint with external plasticisers (dibutyl phthalate, butyl 2-ethylhexyl phthalate, bis(2-ethylhexyl) phthalate and butyl dodecyl phthalate), coalescing agents (butyl benzoate and 2-ethyl-hexyl benzoate) and other additives
**WHITE PAINT SAMPLES**	B3	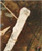	Stick coated with white paint, used to mix paint Taller Sert	C, O, Ti, Na, Al, Si (S, Ca)	TiO_2_ (mainly rutile)	PVAc TiO_2_	acetic acid, acetone, 1,3-cyclopentadiene, benzene (and benzene derivatives), toluene, indane, indene, methylindenes, naphthalene; dibutyl phthalate, butyl 2-ethylhexyl phthalate, bis(2-ethylhexyl) phthalate and butyl dodecyl phthalate; butyl benzoate and 2-ethyl-hexyl benzoate	Pigment: titanium white (titanium dioxide) Extenders: possibly kaolin (hydrated aluminosilicate) Binder: polyvinyl acetate-based paint with external plasticisers (dibutyl phthalate, butyl 2-ethylhexyl phthalate, bis(2-ethylhexyl) phthalate and butyl dodecyl phthalate), coalescing agents (butyl benzoate and 2-ethyl-hexyl benzoate) and other additives
	B4	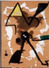	Untitled (FPJM-00135), 1977 Catalogued as oil and acrylic on plywood 122 × 89 cm	C, O, Ti, Na, Al, Si (S, Ca)	TiO_2_ (mainly rutile)	PVAc TiO_2_	acetic acid, acetone, 1,3-cyclopentadiene, benzene (and benzene derivatives), toluene, indane, indene, methylindenes, naphthalene; dibutyl phthalate, butyl 2-ethylhexyl phthalate, bis(2-ethylhexyl) phthalate and butyl dodecyl phthalate; butyl benzoate and 2-ethyl-hexyl benzoate	Pigment: titanium white (titanium dioxide) Extenders: possibly kaolin (hydrated aluminosilicate) Binder: polyvinyl acetate-based paint with external plasticisers (dibutyl phthalate, butyl 2-ethylhexyl phthalate, bis(2-ethylhexyl) phthalate and butyl dodecyl phthalate), coalescing agents (butyl benzoate and 2-ethyl-hexyl benzoate) and other additives
	B6	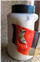	Bottle with white paint, La Pajarita, Son Boter	C and O only	None (amorphous)	PVAc	acetic acid, acetone, 1,3-cyclopentadiene, benzene (and benzene derivatives), toluene, indane, indene, methylindenes, naphthalene; dibutyl phthalate, butyl 2-ethylhexyl phthalate, bis(2-ethylhexyl) phthalate and butyl dodecyl phthalate; butyl benzoate and 2-ethyl-hexyl benzoate; polydimethylsiloxane and silicon oils, siloxanes; ethylene glycol; ammonia acetate	Pigment: none detected Binder: polyvinyl acetate-based paint with external plasticisers (dibutyl phthalate, butyl 2-ethylhexyl phthalate, bis(2-ethylhexyl) phthalate and butyl dodecyl phthalate), coalescing agents (butyl benzoate and 2-ethyl-hexyl benzoate), antifoam agents (siloxanes), freeze–thaw agents (ethylene glycol) and pH buffers (ammonia acetate)

**Table 2:** List of identified compounds in the pyrograms of Figure 3 with corresponding retention times (in minutes) and peak numbers.

Peak Number	RT (min)	Identified Compounds
1	0.969	2-Butene
2	1.065	Acetone
3	1.17	1,3-Cyclopentadiene
4	1.704	Benzene
5	1.755	Acetic acid
6	1.779	Butyl alcohol
7	2.075	Acetic anhydride
8	2.456	Toluene
9	2.738	Acetic acid, butyl ester
10	3.085	Ethylbenzene
11	3.122	p-Dimethylbenzene
12	3.33	Styrene
13	3.544	Benzene, (1-methylethyl)-
14	3.653	Benzene, 2-propenyl-
15	3.704	Benzene, propyl-
16	3.768	Benzaldehyde
17	3.99	Benzene, 2-propenyl-
18	4.173	1-Hexanol, 2-ethyl-
19	4.197	Benzene, 2-propenyl-
20	4.268	Indane
21	4.309	Benzene, 3-butenyl-
22	4.336	Indene
23	4.462	Acetophenone
24	5.105	1,2-Dihydronaphthalene
25	5.234	1,4-Dihydronaphthalene
26	5.401	Naphthalene
27	6.268	α-Methylnaphthalene
28	6.350	Phthalic anhydride
29	6.398	β-Methylnaphthalene
30	6.829	Benzoic acid, butyl ester
31	6.921	Biphenyl
32	7.051	Naphthalene, 1-(2-propenyl)-
33	8.564	Fluorene
34	9.414	Benzoic acid, 2-ethylhexyl ester
35	9.890	Anthracene, 9,10-dihydro-
36	10.050	Anthracene
37	11.132	Dibutyl phthalate
38	13.019	Butyl 2-ethylhexyl phthalate
39	13.373	Phthalic acid, butyl dodecyl ester
40	15.220	Bis(2-ethylhexyl) phthalate

**Table 3:** List of identified compounds in the pyrogram of Figure 4, with corresponding retention times (in minutes) and peak numbers.

Peak Number	RT (min)	Identified Compounds
1	1.262	Propene
2	1.299	1-Butene
3	1.547	1,3-Cyclopentadiene
4	1.877	Acetic acid
5	2.047	Benzene
6	2.105	2-Butenal, (E)-
7	2.265	Ammonium acetate
8	2.289	n-Butyl formate
9	2.646	Benzyl ether
10	2.857	1,3,5-Heptatriene, (E,E)-
11	3.112	Ethylene glycol ethyl ether
12	3.575	2,4-Hexadienal, (E,E)-
13	3.911	Benzaldehyde
14	4.275	Indane
15	4.428	Benzyl alcohol
16	4.592	1,3-Dihydro isobenzofuran
17	4.738	Methyl benzoate
18	5.462	Naphthalene
19	5.904	Benzoic acid
20	6.527	Phthalic acid
21	6.578	Phthalic anhydride
22	6.952	Butyl benzoate
23	7.044	Biphenyl
24	8.047	2-Naphthalenecarboxaldehyde
25	9.459	Ethylhexyl benzoate
26	10.86	Diisobutyl phthalate
27	11.217	Dibutyl phthalate
28	11.462	Butyl octyl phthalate
29	13.077	Phthalic acid, butyl 2-ethylhexyl ester
30	14.523	Butyl isodecyl phthalate
31	15.285	Bis(2-ethylhexyl) phthalate
32	16.281	Phthalic acid, dinonyl ester

## Data Availability

Data collected during the study are available from the authors upon reasonable request.
